# IGF1R and Src inhibition induce synergistic cytotoxicity in HNSCC through inhibition of FAK

**DOI:** 10.1038/s41598-021-90289-1

**Published:** 2021-05-24

**Authors:** Christine E. Lehman, Adam Spencer, Sarah Hall, Jeremy J. P. Shaw, Julia Wulfkuhle, Emanuel F. Petricoin, Stefan Bekiranov, Mark J. Jameson, Daniel Gioeli

**Affiliations:** 1 Department of Otolaryngology-Head and Neck Surgery, University of Virginia School of Medicine, Charlottesville, VA USA; 2 Department of Microbiology Immunology and Cancer Biology, University of Virginia School of Medicine, Charlottesville, VA 22908 USA; 3 Department of Experimental Pathology, University of Virginia School of Medicine, Charlottesville, VA USA; 4 Center for Applied Proteomics and Molecular Medicine, George Mason University, Manassas, VA USA; 5 Department of Biochemistry and Molecular Genetics, University of Virginia School of Medicine, Charlottesville, VA USA; 6 UVA Cancer Center, University of Virginia School of Medicine, Charlottesville, VA USA

**Keywords:** Head and neck cancer, Cell migration, Cell signalling

## Abstract

Head and neck cancer is the sixth most common cancer worldwide with a 5-year survival of only 65%. Targeting compensatory signaling pathways may improve therapeutic responses and combat resistance. Utilizing reverse phase protein arrays (RPPA) to assess the proteome and explore mechanisms of synergistic growth inhibition in HNSCC cell lines treated with IGF1R and Src inhibitors, BMS754807 and dasatinib, respectively, we identified focal adhesion signaling as a critical node. Focal Adhesion Kinase (FAK) and Paxillin phosphorylation were decreased as early as 15 min after treatment, and treatment with a FAK inhibitor, PF-562,271, was sufficient to decrease viability in vitro. Treatment of 3D spheroids demonstrated robust cytotoxicity suggesting that the combination of BMS754807 and dasatinib is effective in multiple experimental models. Furthermore, treatment with BMS754807 and dasatinib significantly decreased cell motility, migration, and invasion in multiple HNSCC cell lines. Most strikingly, treatment with BMS754807 and dasatinib, or a FAK inhibitor alone, significantly increased cleaved-PARP in human ex-vivo HNSCC patient tissues demonstrating a potential clinical utility for targeting FAK or the combined targeting of the IGF1R with Src. This ex-vivo result further confirms FAK as a vital signaling node of this combinatorial treatment and demonstrates therapeutic potential for targeting FAK in HNSCC patients.

## Introduction

Head and neck cancer is the sixth most common cancer worldwide and the ninth most common cancer in the United States with an annual worldwide incidence of approximately 630,000 cases per year^1,2^. Most of these head and neck cancers are squamous cell carcinomas (HNSCC) which arise in the upper aero-digestive tract including the oral cavity, larynx, and oropharynx^2^. These are often functionally and cosmetically devastating diseases due to their location and surgical treatment^3^. While single agent targeted therapies have been tested, these fail to elicit a complete and durable response or significantly improve patient survival. Currently, the 5-year survival for all patients with HNSCC remains around 65%^1^, highlighting the need for an improved understanding of the underlying biologic mechanisms of HNSCC progression and therapeutic response. The incidence of human papillomavirus positive (HPV+) HNSCC overall is increasing. Fortunately, patients with HPV+ HNSCC exhibit improved outcomes with current treatments, whereas there has been little improvement in outcomes for HPV negative (HPV−) HNSCC^4^. Furthermore, resistance to targeted therapies contributes to poor patient outcome and highlights the need to mechanistically understand and combat these events.

Our previous work demonstrated that inhibition of insulin-like growth factor-1 receptor (IGF1R) with BMS754807 in combination with Src family kinase inhibition by dasatinib synergistically inhibits growth of HPV− HNSCC cells in vitro^5^. The importance of IGF1R and Src inhibition for the synergistic growth inhibition was verified in this study through drug substitution experiments using linsitinib to inhibit IGF1R and saracatinib to inhibit Src. The molecular mechanism contributing to this synergistic growth inhibition was not elucidated and could provide insights into novel therapeutic opportunities in HNSCC. The IGF1R was identified as a promising therapeutic target for intervention as it is overexpressed in various cancers and is involved in pro-mitotic and pro-survival signaling^6^. Furthermore, our previous work demonstrates that activation of IGF1R in HNSCC can result in resistance to targeted EGFR inhibitors^7^ and highlights the need for improved mechanistic insight to combat therapeutic resistance. Despite Src overexpression in human HNSCC, suggesting its importance in progression of this disease, a phase II trial of dasatinib alone failed to show efficacy when treating recurrent and/or metastatic HNSCC^8^. The lack of effectiveness from singular IGF1R or Src inhibition suggests that improved molecular understanding could inform combination therapies and may be more efficacious. Treatment of various HNSCC cell lines with IGF1R and Src inhibitors in combination resulted in robust synergy in 8 of the 9 cell lines tested with growth inhibition of at least 49%^5^. This synergistic response among various HPV− HNSCC cell lines with genetic and signaling differences suggests that the IGF1R and Src signaling pathways are critical for survival in a range of HNSCCs. Furthermore, while combined IGF1R and Src inhibition has demonstrated efficacy in various cancer types^5,9–12^, the mechanism for the cytotoxicity to this combination remains under examined and may provide therapeutic insights.

Focal adhesion signaling regulates cellular adhesion, motility, proliferation, and survival in various cells suggesting the importance of inhibiting these pathways in cancer. Focal adhesion kinase (FAK) is also a key factor in control of cell-extracellular matrix interactions and is a key component in growth factor receptor signaling pathways including IGF1R signaling^13,14^. Mechanistically, FAK physically and functionally interacts with Src to promote a multitude of cellular responses in tumor cells including an ability to promote proliferation, tumor metastasis, epithelial mesenchymal transition and anoikis resistance^15^. FAK also interacts directly with IGF1R and this interaction is critical for the growth pancreatic cancer, triple negative breast cancer, and melanoma^16–19^. Furthermore, focal adhesion proteins including FAK are elevated with increased malignancy and invasiveness^20^, and high FAK expression has been implicated in malignant transformation in prostate, breast, colon, head and neck, and thyroid carcinomas^13,21–23^. Increased expression of FAK has also been associated with radioresistance in HNSCC^24^, highlighting the importance of focal adhesion signaling in this disease.

In the present study, we sought to examine the mechanism through which treatment of HNSCC cells with BMS754807 and dasatinib induces synergistic cytotoxicity. Our data identify focal adhesion signaling, particularly inhibition of FAK, as a key molecular event in response to treatment with this drug combination and show that inhibition of FAK alone is sufficient to inhibit growth of HNSCC 3D spheroid cultures and ex-vivo HNSCC patient tissues.

## Materials and methods

### Tissue culture

The HPV- cell lines SCC25, SCC9, Cal27 and FaDu were obtained from ATCC (Manassas, VA) and the HPV− OSC19 cells were generously provided by Dr. Jeffrey Myers (The University of Texas MD Anderson Cancer Center, Houston, TX). All cell lines were grown in DMEM/F-12 media supplemented with 5% fetal bovine serum (FBS) with 400 ng/mL hydrocortisone and maintained in a 37 °C humidified incubator containing 5% CO_2_. Cell line identities were confirmed by DNA fingerprinting (University of Arizona or ATCC).

### Scratch assay

A cell number ranging from 1 to 2 × 10^6^ was plated to ensure approximately 70% confluence in 60 mm plates containing 2 mL of DMEM/F-12 media supplemented with 5% FBS and 400 ng/mL hydrocortisone. Twenty four hours later, the media was then replaced with DMEM/F-12 media supplemented with 0.5% FBS and cultured for 18–24 h. The following day, each plate was scratched at least twice using a 200 µL tip, refreshed with new media containing 0.5% FBS and the appropriate concentration of drug or vehicle. Images were captured at 10× magnification on the EVOS XL Core Cell Imaging System (Invitrogen, Carlsbad, CA) immediately following scratching (0 h) and after 24 h. Wound area was calculated for each scratch using ImageJ.

### Boyden chamber

1.0 × 10^5^ cells were plated in DMEM/F-12 media containing 0.5% FBS in the upper chamber of a transwell with 0.8 µm pores in a total volume of 100 µL with the appropriate concentration of drug(s) or vehicle, as appropriate. The lower chamber contained DMEM/F-12 media containing 20% FBS in a final volume of 600 µL containing the appropriate concentration of drug(s) or vehicle and the cells were then incubated for 24 h. Following incubation, cells were removed from the top of the transwell, and the membrane was cut from the transwell and mounted on a slide with DAPI mounting medium. After allowing the slides to dry, cells were imaged with at least 12 independent fields using an Olympus BX51 microscope and quantified using ImageJ. To assess invasion, the Boyden chamber was coated with 100 µL of Matrigel matrix at a concentration of 250 µg/mL. The transwells were then incubated at 37 °C for 2 h, remaining liquid was removed from the membrane, and the cells were then plated, drugged, and analyzed as described above.

### Reverse phase protein array construction and analysis

Pathway activation mapping was performed by reverse phase protein microarray (RPPA) as previously described^25–27^. Briefly, cells were subjected to lysis with 2.5% solution of 2-mercaptoethanol (Sigma, St. Louis, MO) in Tissue Protein Extraction Reagent (t-PER™ Pierce)/2X SDS Tris-Glycine buffer (Invitrogen, Carlsbad, CA). The lysates were printed in triplicate on glass-backed nitrocellulose array slides (Grace Bio-Labs, Bend, OR) using an Aushon 2470 arrayer (Aushon BioSystems, Burlington, MA) equipped with 185 µm pins. Arrays were blocked (I-Block, Applied BioSystems, Foster City, CA) for 1 h and subsequently probed with primary antibodies. Detection was performed using a fluorescence-based tyramide signal amplification strategy using Streptavidin-conjugated IRDye680 (LI-COR Biosciences, Lincoln NE) as detection reagent. All antibodies were validated for single band specificity as well as for ligand-induction (for phospho-specific antibodies) by immunoblotting prior to use on the arrays as described previously^25–27^. Each array was scanned using a TECAN LS laser scanner (Tecan, Durham NC). After scanning, spot intensity was analyzed, data were normalized to total protein and a standardized, single data value was generated for each sample on the array by MicroVigene software V2.999 (VigeneTech, North Billerica, MA) as previously described^25^. Changes in protein and phospho-protein expression across various time points and cell lines were then analyzed using the software program “R”. Significant synergistic changes in protein and phospho-protein levels were defined as changes induced by the combination treatment minus half the change from each single drug with a false discovery rate of less than 5 percent based on the Bliss model of independence^28^.

### Immunoblot

Cells were plated in DMEM/F-12 media containing 5% FBS and 400 ng/mL hydrocortisone on 60 mm plates, allowed to incubate for 18 h at 37 °C; the media was refreshed, and drug(s) or vehicle were added for 3 h as appropriate. Cells were washed with ice cold PBS containing 2 mM sodium orthovanadate and lysed in a triton lysis buffer containing phosphatase and protease inhibitors. A BCA protein assay was used to determine protein concentration and a SDS-PAGE was then performed as previously described^7^. Proteins were blotted and transferred onto nitrocellulose. Membranes were blocked and antibodies were diluted with TBST containing 3% BSA. Proteins were visualized using the Odyssey imaging system (LICOR Biosciences, Lincoln, NE) and densitometry was quantified using Image Studio software (LICOR Biosciences, Lincoln, NE). Antibodies used: FAK Y397 (Cell signaling, #3283S), FAK (Cell signaling, #3285), Ran (Cell signaling, #4462), Src (Cell signaling, #2108), Src Y416 (Cell signaling, #6943), Paxillin Y118 (Cell signaling, #2541), Paxillin (BD Transduction, #610052), IGF1Ra (Cell signaling, #3018), IGF1R Y1135/Y1136 (Cell signaling, #3024), Actin (Santa Cruz, #sc47778). Proteins were normalized for quantification using at least 2 loading controls for each respective membrane. Total and phospho primary antibodies to the same target protein were combined and probed on the same membrane if they were from a difference species.

### CyQUANT

Cells were plated in 96-well plates at a density of 5000 cells per well in DMEM/F12 containing 0.5% FBS. The following day, cells were treated for 72 h with the various drug(s), then the media was then aspirated and 50 μL of CyQUANT solution was added to each well, incubated at 37 °C for 45 min and plates were analyzed using a BioTek Synergy plate reader (BioTek, Winooski, VT).

### Clonogenic assay

Cells were plated in 6-well plates at a density of 250 cells per well in DMEM/F12 containing 5% FBS. The following day, cells were treated with vehicle or various drug combinations for 5 days. The media was then replaced with fresh media with or without drugs and the cells were allowed to grow for another 4 days. Cells were fixed with 10% buffered formalin for 15 min followed by staining with 0.1% crystal violet^29^. Cells were then imaged and quantified using ImageJ.

### 3D spheroid culture

2000 cells per well were plated in 96-well round bottom ultra-low adherent tissue culture plates in 180 µL of media. Cells were treated 48 h later with vehicle or various drug combinations. Cells were incubated for 7 days in typical cell culture conditions and cell viability was then analyzed using ATPlite 3D. ATPlite 3D was chosen over AlamarBlue or CyQUANT due to its improved dynamic range and reproducibility when used with the 3D cultures. Drug concentrations were selected based on dose response experiments previously performed in all 3 cell lines. For each combination in each cell line, we determined the growth inhibition caused by each drug alone as well as 9 combinations (3 concentrations of drug A combined with 3 concentrations of drug B). The results were then compared to a predicted growth inhibition generated using the Bliss model of additivity^30^ to determine whether the drug interaction was synergistic, additive, or antagonistic.

### Ex vivo culture

Patient ex vivo cultures were established as has been done for HNSCC and a variety of solid tumors^33–33^. Samples of human HNSCC tumors were collected immediately after surgical tumor resection from a portion of the tumor deemed to be non-critical for clinical pathologic analysis. Tumor samples were de-identified and received under a tissue banking protocol which does not require informed consent and was approved by the University of Virginia Institutional Review Board for Health Sciences Research (IRB-HSR #13457). All methods involving these tumors were carried out in accordance with relevant IRB guidelines and regulations. Specimens were transported in saline and subsequently cultured in RPMI1640 containing 15% FBS, 1X MEM nonessential amino acids, 1X amphotericin B (Fungizone), 1X sodium pyruvate and 1X gentamicin. Before culturing, the tumors were washed twice with PBS, cut into approximately 2 × 2 × 1 mm blocks, and placed on rehydrated Gelfoam (VWR, Radnor, PA) in 60 mm dishes containing the appropriate drug(s) or control. Three tissue pieces were placed on each Gelfoam and incubated at 37 °C for 3 days with or without drugs, then placed in 1 mL of 10% buffered formalin overnight and then placed into 70% ethanol at 4 °C before embedding.

### Tissue cancer genome atlas (TCGA) analysis

mRNA sequencing data was downloaded from the TCGA database within the “Head and Neck Squamous Cell Carcinoma, PanCancer Atlas^34^”. The “Reads Per Kilobase of transcript per Million mapped reads” (RPKM) were obtained for PTK2 (FAK), PTK2B, and PAX genes. To create the “all samples” analyses, the RPKM values of all tumor samples (523) and all normal samples (44) were plotted and statistically analyzed using a non-paired T test. For the “matched samples” analyses, the 43 patients that had both “normal” and “tumor” samples were analyzed. The RPKM of each patient’s normal tissue sample was compared to that same patient’s tumor sample value. These values were then plotted and statistically analyzed using a paired T test.

### Statistical analysis

All statistical analyses were performed using GraphPad Prism version 8. Data was analyzed using one-way analysis of variance (ANOVA) with post-hoc Tukey to test for differences.

## Results

### Treatment with BMS754807 and dasatinib significantly alters focal adhesion signaling

**Figure 1:**
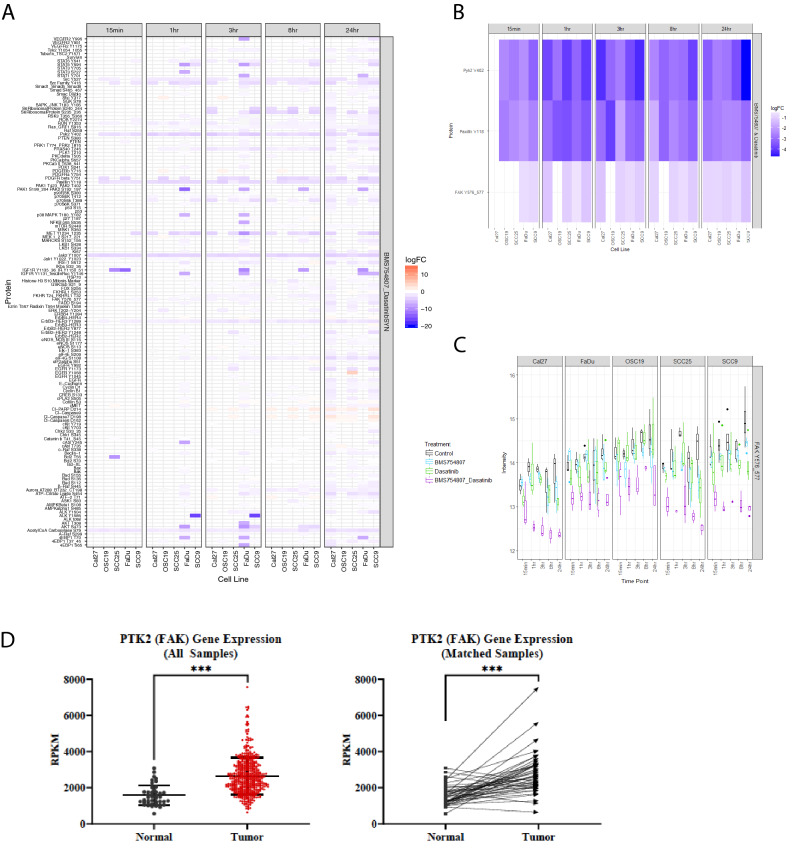
Focal adhesion proteins that are upregulated in HNSCC are synergistically decreased with BMS754807 and dasatinib. Lysates from five HNSCC cell lines were assessed by reverse phase protein array (RPPA) to determine protein changes following treatment with vehicle, each single drug, or the combination at 15 min, 1 h, 3 h, 8 h and 24 h. Synergistic changes are defined as “Log Fold Change (LogFC) in combination—(1/2LogFC Drug 1)—(1/2LogFC Drug 2)” with a false discovery rate (FDR) < 0.05. (**A**) Representative heat map depicting all synergistic changes among the 145 epitopes assessed by RPPA. (**B**) Heat map depicting synergistic changes in the focal adhesion proteins Pyk2 Y402, Paxillin Y118 and FAK Y576-577. (**C**) Graphs depict changes in protein expression from treatment with control, BMS754807, dasatinib or BMS754807 and dasatinib combination. (**D**) mRNA expression in tumor or normal head and neck tissues from the TCGA panCancer Atlas. The “All Samples” graph depicts the reads per kilobase of transcript per million mapped reads (RPKM) value from 522 tumor samples and 44 normal samples. The “Matched samples” compares the RPKM of 43 patients with both tumor and normal sample.

To determine the mechanism through which treatment of HNSCC cell lines with BMS754807 and dasatinib induces synergistic cytotoxicity, a panel of 5 cell lines at 5 time points ranging from 15 min to 24 h post-treatment were analyzed by reverse phase protein array (RPPA) for the level of 145 proteins and phosphoproteins. To analyze these 3625 RPPA data points, the *limma* package in “R” was used to visualize changes in protein and phosphoprotein levels and assess synergistic changes (Fig. 1A). Significant synergistic changes in proteins and phosphoproteins were defined as changes induced by the combination treatment minus half the change from each single drug with a false discovery rate of less than 5 percent based on the Bliss model of independence^28^. Three proteins involved in focal adhesion signaling, Pyk2, paxillin, and FAK, demonstrated synergistic decreases across nearly all time points and cell lines analyzed (Fig. 1B), suggesting their importance in the mechanism of cytotoxicity induced by combined inhibition of IGF1R and Src. We next analyzed the contribution of each single drug to the changes identified in Fig. 1A,B. Figure 1C represents this analysis for FAK phospho-Y576-577. Paxillin, and Pyk2 are shown in Supplemental Fig. 1. As demonstrated, despite limited effect of BMS754807 or dasatinib alone on FAK phospho-Y576-577 levels, the combined inhibition of IGF1R and Src significantly decreased FAK phospho-Y576-577 levels, suggesting FAK is a vital node of convergence between the IGF1R and Src pathways. To examine the relevance of FAK in HNSCC, we analyzed HNSCC data from The Cancer Genome Atlas (TCGA). FAK (PTK2) is amplified or mutated in approximately 26% of HNSCC suggesting its importance in the pathogenesis of this disease. Strikingly, further analysis of data within the TCGA database demonstrates significantly higher FAK (PTK), Pyk2 (PTK2B), and paxillin (PAX) mRNA in tumor samples compared to normal (Fig. 1D; Supplemental Fig. 2). Furthermore, when matched tumor and normal tissues were available (44 total patients), mRNA levels were significantly increased in matched tumor sample as compared to normal (Fig. 1D; Supplemental Fig. 2), indicating the importance of focal adhesion signaling in the pathology of HNSCC.

**Figure 2:**
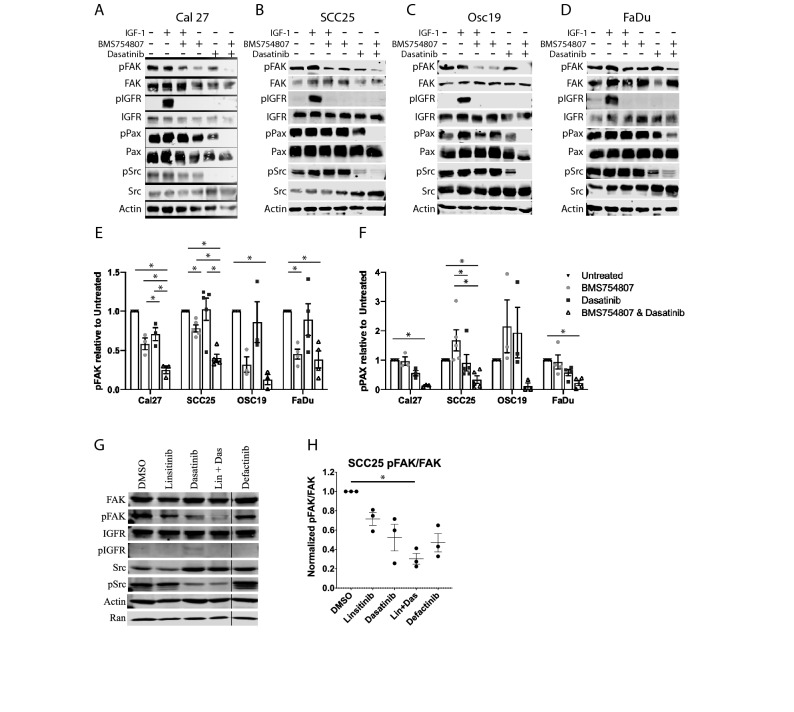
Treatment with BMS754807 and dasatinib alter focal adhesion signaling. Cal27 (**A**), SCC25 (**B**), OSC19 (**C**), FaDu (**D**) cells were treated with 1 µM BMS754807 ± 10 nM des(1–3)IGF-1, 25 nM dasatinib, or the combination of 1 µM BMS754807 and 25 nM dasatinib as indicated and immunoblot was performed. Representative blots from 3 to 5 independent experiments are shown. Each section of the membrane was probed independently for the respective labeled antibody and imaged independently when necessary to insure appropriate exposure. Each cell line was probed independently, cropping is delineated by white spacing between epitopes or black vertical line. Uncropped western blot images are shown in Supplemental Fig. 8. Changes in FAK Y397 (**E**) and paxillin Y118 (**F**) are quantified relative to untreated and represent the mean and SEM of 3–5 independent experiments. (**G**) SCC25 cells treated with 250 nM linsitinib or 25 nM dasatinib singly or in combination as in (**A**–**E**) and (**H**) quantified relative to untreated and represent the mean and SEM of 3 independent experiments. Asterisks represent p < 0.05 as determined by one-way ANOVA and Tukey post-hoc analysis.

To verify the results demonstrated by RPPA analysis, we used immunoblot to examine changes in phosphorylation of FAK and paxillin across Cal27, SCC25, Osc19, and FaDu after 3 h of treatment with either vehicle, BMS754807 alone, dasatinib alone, or the combination of BMS754807 and dasatinib. Stimulation with IGF1 with and without BMS754807 was used as a control to ensure the effectiveness of BMS754807; treatment of IGF1-stimulated cells with BMS754807 returned IGF1R phosphorylation to unstimulated levels (Fig. 2A–D). As shown in Fig. 2A–D, treatment with the combination of BMS754807 and dasatinib reduced phosphorylation of FAK Y397 and paxillin Y118 compared to untreated cells. Furthermore, as quantified in Fig. 2E,F, phosphorylation FAK Y397 was significantly lower in Cal27 and SCC25 than treatment with the single drugs alone, confirming the findings of the RPPA. For Osc19, only the combination of BMS754807 and dasatinib showed significant pY397 FAK inhibition compared to control, and in FaDu both the combination of BMS754807 and dasatinib, and BMS754807 alone significantly inhibited FAK Y397 phosphorylation. These data are consistent with the RPPA data showing that FAK activity is inhibited in the drug combination to a greater extent than either drug alone. Since there are concerns of drug selectivity, we analyzed the drugs used herein using the proteomicsDB resource^37–38^. According to the proteomicsDB, BMS754807 preferentially inhibits IGF1R but has significant activity against FAK, and dasatinib has many well-known targets in addition to Src (Supplemental Fig. 3). Thus, some of the effect of the BMS754807 and dasatinib combination on pY397 FAK levels may be due to direct inhibition of FAK by BMS754807. At the BMS754807 concentration used, FAK inhibition was only observed in SCC25 and FaDu cells, and in SCC25 cells the combination was more effective at inhibiting FAK than BMS754807 or dasatinib alone. The importance of FAK downstream of IGF1R and dasatinib was confirmed in SCC25 cells using linsitinib to inhibit IGF1R where the combination of linsitinib and dasatinib significantly inhibited pY397 FAK (Fig. 2G,H). Collectively the data suggest the importance of focal adhesion signaling in the synergistic cytotoxicity of IGF1R and Src inhibition.

### Inhibition of FAK is sufficient to reduce cell viability in HNSCC cells

**Figure 3:**
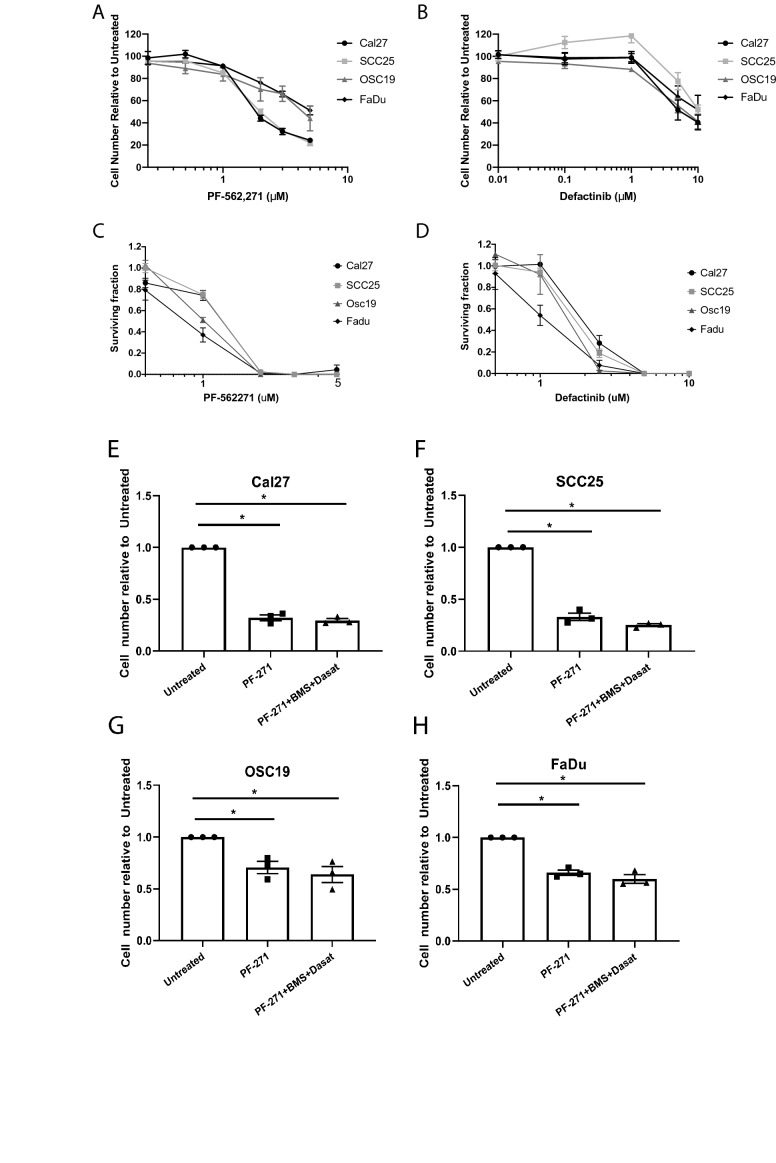
Treatment with BMS754807 and dasatinib decreases viability through a FAK dependent mechanism. Indicated cell lines were treated with varying concentrations of PF-562,271 (**A**) or defactinib (**B**) for 72 h in 2D culture followed by quantification using CyQUANT. (**C**,**D**) cell lines were treated with varying concentrations of PF-562,271 (**C**) or defactinib (**D**) for 9 days in a clonogenic assay followed by quantification crystal violet staining. Cal27 (**E**), SCC25 (**F**), OSC19 (**G**), and FaDu (**H**) cells were treated with PF-562,271 (PF) alone or in combination with BMS754807 (BMS) and dasatinib (Dasat) followed by CyQUANT assay to determine relative cell number. Data represents the mean ± SEM of 3 independent replicates for each cell line. Asterisks represent p < 0.05 as determined by one-way ANOVA and Tukey post-hoc analysis.

To determine whether FAK inhibition alone is sufficient to inhibit HNSCC cell viability, and thereby support its importance as a critical node in HNSCC, four HNSCC cell lines were treated with two independent FAK inhibitors for 72 h and cell viability was assessed using CyQUANT. As established in Fig. 3A, treatment with PF-562,271 decreased cell number 49–78% at a concentration of 5 µM. Treatment with defactinib also decreased cell number 37–60% at a dose of 10 µM (Fig. 3B). We next determined the effect of FAK inhibition on colony forming ability using a clonogenic cell assay^29^ (Fig. 3C,D; Supplemental Fig. 4). Both PF-562,271 and defactinib robustly inhibited colony formation with 1 µM PF-562,271 and 5 µM defactanib completely preventing colony formation. Thus, FAK inhibition is sufficient to reduce viability of HNSCC cells in vitro*.*

To further address the role of FAK inhibition in combined IGF1R and Src-targeted cytotoxicity with BMS754807 and dasatinib, cells were treated with PF-562,271 alone followed by the combination treatment with BMS754807 and dasatinib. If combined BMS754807 and dasatinib treatment causes cytotoxicity through FAK inhibition, prior FAK inhibition by PF-562,271 should prevent additional cytotoxicity. As shown in Fig. 3E–H, treatment with 3 µM PF-562,271 alone significantly decreases cell viability; subsequent addition of BMS754807 and dasatinib did not result in additional significant cytotoxicity. These data demonstrate that FAK inhibition is sufficient to reduce cell viability and further support that combination treatment with BMS754807 and dasatinib causes cytotoxicity via a FAK-dependent mechanism.

### Treatment with BMS754807 and dasatinib reduces motility, migration, and invasion of HNSCC cells

**Figure 4:**
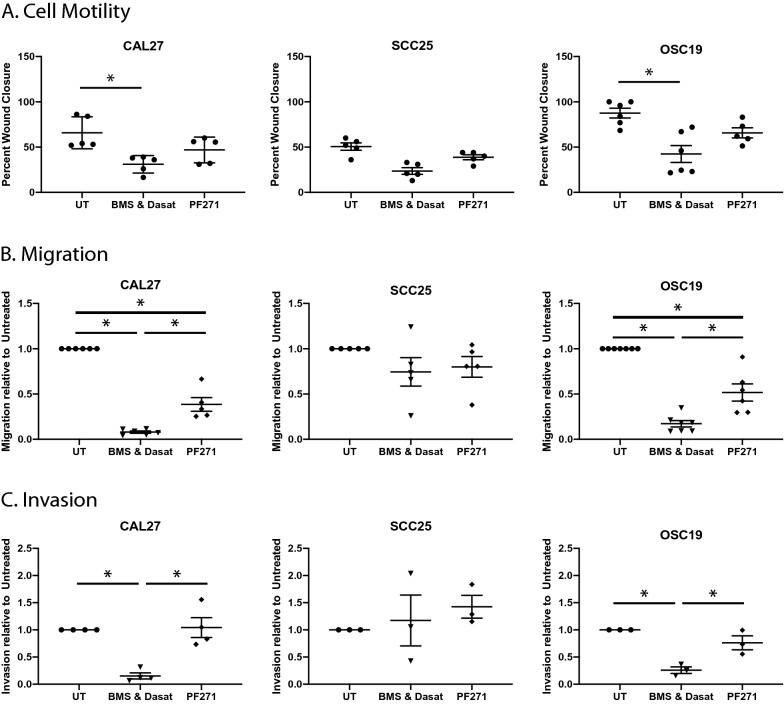
Treatment with BMS754807 and dasatinib decreases motility, migration and invasion of HNSCC. (**A**) Graphs represent mean wound closure ± SEM in each cell line. (**B**) Graphs represent relative migration of cells through a Boyden chamber as compared to untreated cells. (**C**) Relative invasion of HNSCC through a Matrigel-coated Boyden chamber as compared to untreated cells. Quantification by ImageJ. Data is depicted by representation for each independent replicate and the mean ± SEM of at least 5 independent replicates. Asterisks represent p < 0.05 as determined by one-way ANOVA and Tukey post-hoc analysis.

A defining feature of malignancy is the ability to invade surrounding tissues and to metastasize. Furthermore, metastatic disease is responsible for over 90% of cancer-related mortality^39^ demonstrating the importance of understanding and inhibiting this process. As FAK has an established critical role in the process of cell motility, migration, and invasion^20,40,41^, we investigated whether treatment of HNSCC cells with the combination of BMS754807 and dasatinib could reduce motility, migration and/or invasion by using scratch assay and Boyden chamber assays. To investigate motility, Cal27, SCC25, and OSC19 cell lines were examined in scratch assays. As shown in Fig. 4A, the combination treatment significantly reduced motility relative to untreated in Cal27 and OSC19 cells. Representative images of cells after 24 h incubation are shown in Supplemental Fig. 5. Cells treated with PF-562,271 also showed a slight decrease in motility across the cell lines, although to a lesser degree than combined treatment with IGF1R and Src inhibitors.

To examine migration of these cells, a Boyden chamber assay was used. Cells were plated on top of a membrane with 8 µm pores and allowed to migrate toward a chemoattractant on the other side of the membrane. The same three cell lines examined by scratch assay were further examined with this technique. Representative images of cells after 24 h incubation are shown in Supplemental Fig. 6. The combination treatment of BMS754807 and dasatinib and the FAK inhibitor PF-562,271 significantly reduced migration in Cal27 and OSC19 cells when compared to untreated cells (Fig. 4B). These data suggest the utility of BMS754807 and dasatinib in the inhibition of migration and metastasis.

As FAK has been implicated in invasion^42–45^, a Boyden chamber was used to examine the effect of combination treatment on invasion of HNSCC through Matrigel. Cells were plated above a membrane containing 8 µm pores that had been previously coated with Matrigel as an extracellular matrix. Treatment of Cal27 and OSC19 cells with the BMS754807 and dasatinib combination significantly decreased invasion compared to untreated cells (Fig. 4C). Representative images of cells after 24 h incubation are shown in Supplemental Fig. 7. Unexpectedly, treatment with PF-562,271 did not significantly alter invasion compared to untreated cells, suggesting alteration in focal adhesion signaling is not sufficient to alter invasion in HNSCC. This demonstrates that combined inhibition of IGF1R and Src with BMS754807 and dasatinib may be more effective at inhibiting invasion then FAK alone, possibly due to the effects of inhibiting upstream targets or the other molecular targets of BMS754807 and dasatinib.

### BMS754807 and dasatinib treatment causes cytotoxicity in 3D spheroid culture

**Figure 5:**
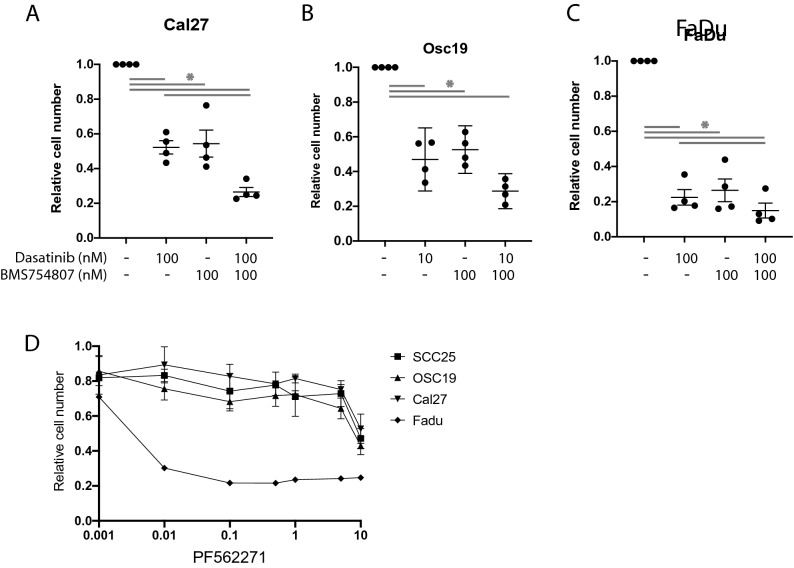
Inhibition of FAK, IGF1R and Src induces cytotoxicity in HNSCC spheroids. (**A**–**C**) Efficacy of BMS754807 and dasatinib on Cal27, Osc19, and FaDu on tumor spheroids following 7 days treatment. Data is presented as the mean ± SEM of 3–4 independent biological replicates. (**D**) PF-562,271 dose response of tumor spheroids. Asterisks represent p < 0.05 as determined by one-way ANOVA and Tukey post-hoc analysis.

To explore the efficacy of the BMS754807 and dasatinib combination in more sophisticated models of HNSCC we generated 3D spheroids from Cal27, FaDu, and OSC19 cells through culture in ultra-low adherent cell culture plates. Tumor spheroids were treated with BMS754807 and dasatinib. Cell viability was assessed using ATPlite after seven days of drug treatment. In this assay, combined BMS754807 and dasatinib treatment induced additive cytotoxicity according to the Bliss model of independence. For Cal27, Osc19, and FaDu, BMS754807 and dasatinib significantly inhibited tumor spheroid growth, as did the single drug treatments of BMS754807 or dasatinib (Fig. 5A–C). The efficacy of FAK inhibition on tumor spheroid growth was tested next using multiple doses of PF-562,271 on Cal27, SCC25, Osc19, and FaDu tumor spheroids. Cal27, SCC25, and Osc19 reached an IC_50_ at 10 μM and FaDu was exquisitely sensitive to PF-562,271 with an IC_50_ of less than 10 nM (Fig. 5D). These data suggest that FAK inhibition or dual treatment with BMS754807 and dasatinib can inhibit HNSCC growth with differential sensitivities.

### BMS754807 and dasatinib or PF-562,271 treatment induces apoptosis in ex vivo HNSCC tumor explants

**Figure 6:**
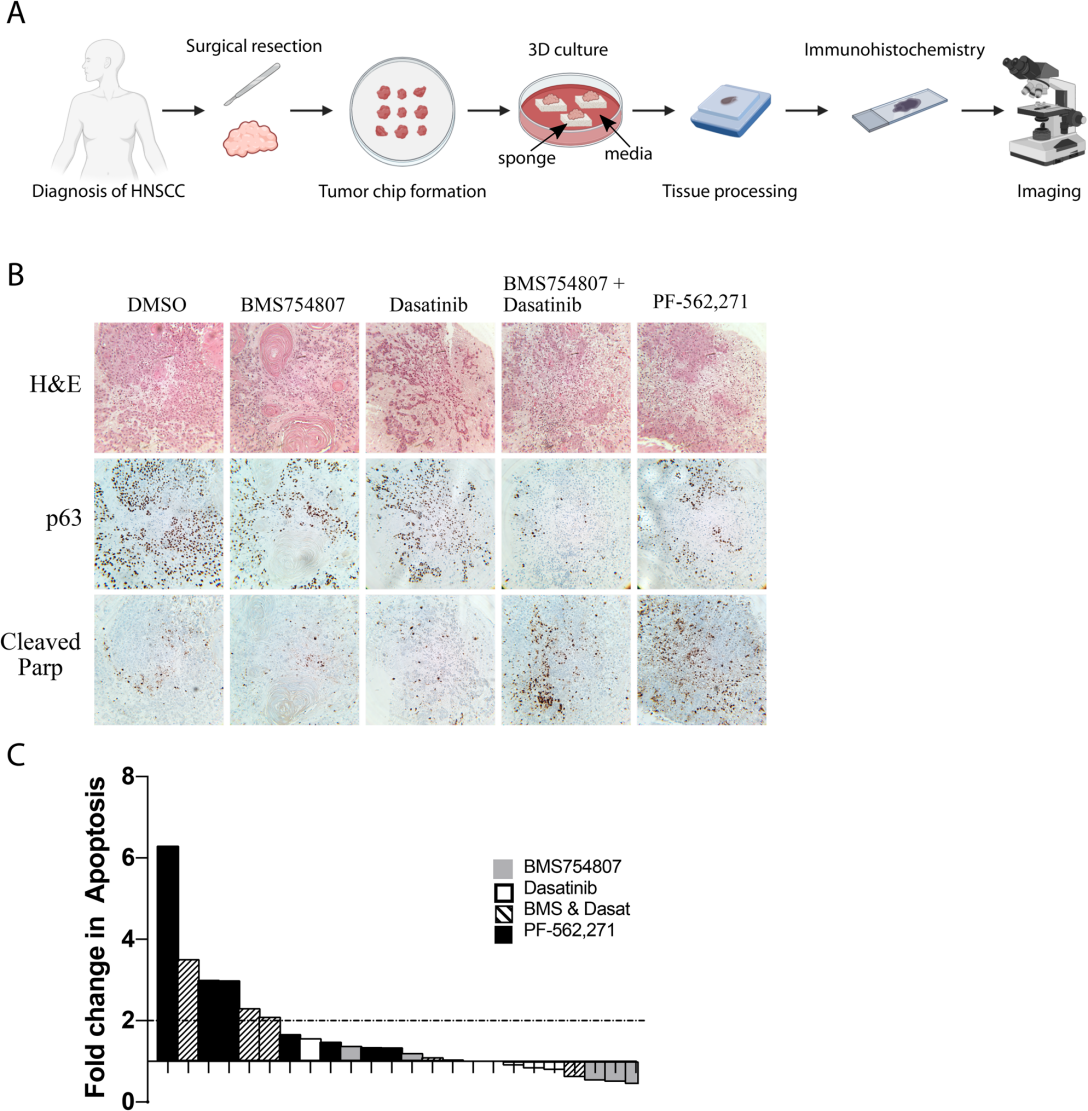
Treatment of ex vivo HNSCC tumor tissue with PF-562,271 or BMS754807 and dasatinib induces significant apoptosis. (**A**) Representation of the work-flow used to analyze human HNSCC tumor ex vivo tissue samples. The image was created with BioRender.com. (**B**) Representative histology images stained as indicated. (**C**) Graph represents the fold change in apoptosis as determined by cleaved-PARP staining in each of 6 ex vivo HNSCC tissue samples treated with either BMS754807, dasatinib, BMS754807 and dasatinib or PF-562,271.

Ex vivo cultures of HNSCC tumor samples taken directly from patients were used to assess the efficacy of the FAK inhibitor PF-562,271 or the combination of BMS754807 and dasatinib (Fig. 6A). Ex vivo explant systems preserve the tumor microenvironment by histologic analysis and are an effective platform to evaluate drug response^46^. Six human HNSCC tumor specimens from patients ranging in age from 40 to 84 years old with tumors from various anatomical sites with varying stage disease were included for analysis (see Supplemental Table 1 for patient information). Immediately following surgical resection, tumor specimens were minced into 2 × 2 × 1mm pieces and cultured on surgical sponges as has been done previously for HNSCC and other solid tumors^31–33^. Ex vivo tumor chips were cultured for 3 days with or without drug(s). Following drug treatment, tumor chips were embedded and processed for hematoxylin and eosin staining and immunohistochemistry with antibodies specific for p63 and cleaved-PARP (Fig. 6B). Quantification of cleaved-PARP positive cells demonstrated three patients with significant induction of apoptosis (> 2-fold) upon treatment with PF-562,271 or BMS754807 and dasatinib (Fig. 6C). These results demonstrate sensitivity to FAK inhibition in some HNSCC tumors and also further demonstrate the potential clinical utility of combined IGF1R and Src inhibition for a subset of patients.

## Discussion

The promise of targeted therapy is that identification of the genetic changes underlying cancer initiation and progression and the rational development of drugs that target those changes will yield effective treatments with minimal toxicity. However, single agent therapies targeting signal transduction pathways have generally yielded disappointing results with responses that are usually partial or not durable. The IGF1R is a known resistance mechanism involved in several treatment approaches for a variety of cancers including HNSCC^47–50^. While HPV+ patients exhibit improved outcomes with current treatments, improved therapeutic options are crucial for the treatment of HPV− HNSCC^4^. It is therefore critical to understand mechanisms through which combinatorial therapies that include IGF1R inhibition induce synergistic cytotoxicity and to identify critical nodes of convergence between the primary drug targets. This knowledge may enable identification of additional therapeutic targets and to more appropriately combine therapies in order to inhibit cancer cell proliferation, invasion and metastasis.

Our prior work demonstrates that treatment of HPV− HNSCC cell lines with the combination of an IGF1R inhibitor, BMS754807, and a Src family kinase inhibitor, dasatinib, induces synergistic cytotoxicity^5^. In the present study, we identify and validate a significant decrease in focal adhesion signaling following treatment with BMS754807 and dasatinib and go on to show that FAK inhibition is sufficient to inhibit HNSCC cell growth in 2D and 3D systems and patient ex vivo cultures. FAK inhibition is sufficient to increase apoptosis in three of six ex vivo HNSCC tumors providing further evidence for FAK as a HNSCC therapeutic target. Interestingly, the three patient tumors that respond to PF-562,271 also responded to combined BMS754807 and dasatinib, further supporting the concept that FAK is a critical node downstream of IGF1R and Src inhibition. In TCGA PanCancer Atlas, PTK2 (FAK) is overexpressed in approximately 25% of HNSCC tumors and FAK mRNA is significantly upregulated in tumor versus normal tissues, further highlighting a role for FAK in HNSCC. In addition to FAK, Pyk2 and paxillin expression is also elevated, indicating that focal adhesion signaling is elevated in HNSCC. Considering the cancer-specific increase in FAK expression as well as our results demonstrating a decrease in cell viability upon FAK inhibitor treatment, FAK emerges as a viable therapeutic target for HNSCC. Other studies have implicated FAK in cell survival and proliferation of HNSCC. Treatment with the FAK inhibitor, TAE226, was shown to induce dormancy in laryngeal SCC cells^51^, siRNA inhibition of FAK induced decreased proliferation in oral squamous cell carcinoma cells^52^, and elevated FAK expression in slow-growing SCC25 cells caused an increase in cell growth^53^. FAK is also implicated in therapeutic resistance in HNSCC. FAK protein and mRNA are upregulated in HPV- HNSCC cells and FAK inhibition with PF-562,271 leads to radiosensitization when cells were treated with 2, 4, or 6 gy^24^. Furthermore, HNSCC grown in 3D were radiosensitized by treatment with the FAK inhibitor, TAE226, or siRNA targeting FAK^54,55^. Overexpression of wild-type or catalytically active FAK was also shown to increase clonogenic survival following radiation^56^, demonstrating an essential role of FAK signaling in resistance to radiotherapy in HNSCC. However, one previous study did not find a significant alteration in proliferation of SCC-40 and SCC-38 HNSCC cells upon FAK inhibition and is in discrepancy with our findings reported here^57^. Both of these lines harbor TP53 inactivating mutations whereas the cell lines used in our study have TP53 gain of function activations^58^. This would suggest a possible dependence on FAK for proliferation when p53 acquires dominant-negative activities, although further studies are necessary to test this hypothesis.

High FAK expression levels have been implicated in malignant transformation in multiple cancers including prostate, breast, colon, thyroid carcinomas, and HNSCC^13,21–23^. FAK is a predictive biomarker for radioresistance in HNSCC^59^ and promotes invasion and migration^20,21,40,45,60,61^, suggesting that FAK inhibition may be useful to combat resistance as well as migration and invasion. FAK is also an independent predictor of nodal metastasis in HNSCC with significantly worse metastasis-free survival if any FAK expression was observed^62^. Furthermore, FAK activation is required for IGF1R-mediated regulation of migration and invasion in triple negative breast cancer cells^18^. FAK and IGF1R interaction has also been shown to be critical for melanoma tumor cell growth^19^. For these reasons, FAK inhibitors have reached clinical trials including GSK2256098, VS-4718, PF-562,271 (VS-6062), defactinib (VS-6063), and BI853520^63^. PF-562,271 was discontinued for non-linear pharmacokinetics in favor of defactinib, which is being used for ongoing clinical trials^64–66^.

Five-year survival rates for stage III and IV HNSCC remain below 50% despite improvements in treatment^67^. There is significant value in identifying combinatorial therapies to prevent or overcome resistance and provide durable therapeutic responses in patients. We have shown that co-targeting IGF1R and Src with BMS754807 and dasatinib in HNSCC cells synergistically inhibits growth in both 2D^5^ and 3D in vitro models and inhibits migration and invasion. Furthermore, we demonstrated that FAK inhibition is a critical node of convergence in the response to combined IGF1R and Src inhibition with these compounds. Importantly, we have shown significant response to the FAK inhibitor PF-562,271 alone, or the combination of BMS754807 and dasatinib in patient ex vivo human HNSCC tumors suggesting that potential exists for targeting FAK, IGF1R, and Src in HNSCC.

## Supplementary Information

Supplementary Information 1.
