# Supplementary material for: IGF1R and Src inhibition induce synergistic cytotoxicity in HNSCC through inhibition of FAK
Source: Sci Rep. 2021 May 24;11:10826. doi: 10.1038/s41598-021-90289-1 (PMC8144381; doi:10.1038/s41598-021-90289-1)
Supplement: Supplementary file 1 — Supplementary Information 1. [file 41598_2021_90289_MOESM1_ESM.pdf]

IGF1R and Src inhibition induce synergistic cytotoxicity in HNSCC through inhibition of FAK

Christine E Lehman, PhD[1]; Adam Spencer[1]; Sarah Hall[1]; Jeremy JP Shaw[3]; Julia Wulfkuhle, PhD[4]; Emanuel F Petricoin, PhD[4]; Stefan Bekiranov, PhD[5]; Mark J Jameson, MD, PhD[1,6]; Daniel Gioeli, PhD[2,6*]

Departments of Otolaryngology – Head & Neck Surgery[1], Microbiology Immunology & Cancer Biology[2], Experimental Pathology[3], Biochemistry and Molecular Genetics[5], and UVA Cancer Center[6], University of Virginia School of Medicine, Charlottesville, VA

Center for Applied Proteomics and Molecular Medicine[4]

George Mason University, Manassas, VA

* To whom correspondence should be addressed. Daniel Gioeli, Department of Microbiology, Immunology, and Cancer Biology, University of Virginia, Charlottesville, Virginia, 22908, United States of America; Tel: (1) 434-982-4243; Fax: (1) 434-982-0689; Email: dgioeli@virginia.edu

Running title: IGF1R and Src inhibition induce cytotoxicity through FAK

Supplemental Figure 1. BMS754807 and dasatinib treatment induces a synergistic decrease in activation of the focal adhesion proteins paxillin and Pyk2. Graphs depict log fold changes in expression of (A) Paxillin Y118 and (B)Pyk2 Y402 from treatment with control, BMS754807, dasatinib or BMS754807 and dasatinib combination.

Supplemental Figure 2. PTK2B and PXN are overexpressed in HNSCC tumor tissues. mRNA expression or (A) PTK2B (Pyk2) and (B) PXN (Paxillin) in tumor or normal head and neck tissues from the TCGA panCancer Atlas The "All Samples" graph depicts the reads per kilobase of transcript per million mapped reads (RPKM) value from 522 tumor samples and 44 normal samples. The "Matched samples" compares the RPKM of 43 patients with both tumor and normal sample.

Supplemental Figure 3: Drug target inhibition profiles from proteomicsDB . The drug target inhibition profiles for BMS754807, dasatinib, PF562271, Linsitinb, and defactinib from proteomicsDB for relevant targets are shown.

Supplemental Figure 4: Clonagenic assay images. Shown are images from representative clonagenic assays quantified in Figure 3C, D.

Supplemental Figure 5. BMS754807 and dasatinib treatment decreases motility of HNSCC cells. Representative images of wound areas 24 hours following scratch and respective treatment of Cal27, SCC25, and OSC19 cells.

Supplemental Figure 6: BMS754807 and dasatinib treatment decreases migration of HNSCC cells. Representative images of migrated cells stained with DAPI 24 hours following plating and treatment of Cal27, SCC25, and OSC19 cells.

Supplemental Figure 7: BMS754807 and Dasatinib treatment decreases invasion of HNSCC cells. Representative images of invaded cells stained with DAPI 24 hours following plating and treatment of Cal27, SCC25, and OSC19 cells.

Supplemental Figure 8: Western blot analysis of HNSCC lines treated with BMS754807 and dasatinib. Shown are uncropped images from the western blots shown and quantified in Figure 2. Red brackets represent cropped areas.

Supplemental Table 1: Characteristics of six HNSCC patient samples analyzed in *ex-vivo* culture. Six tumors were removed from various anatomical locations by surgical resection from patients with differing age and disease stage.

Supplemental Figure 1

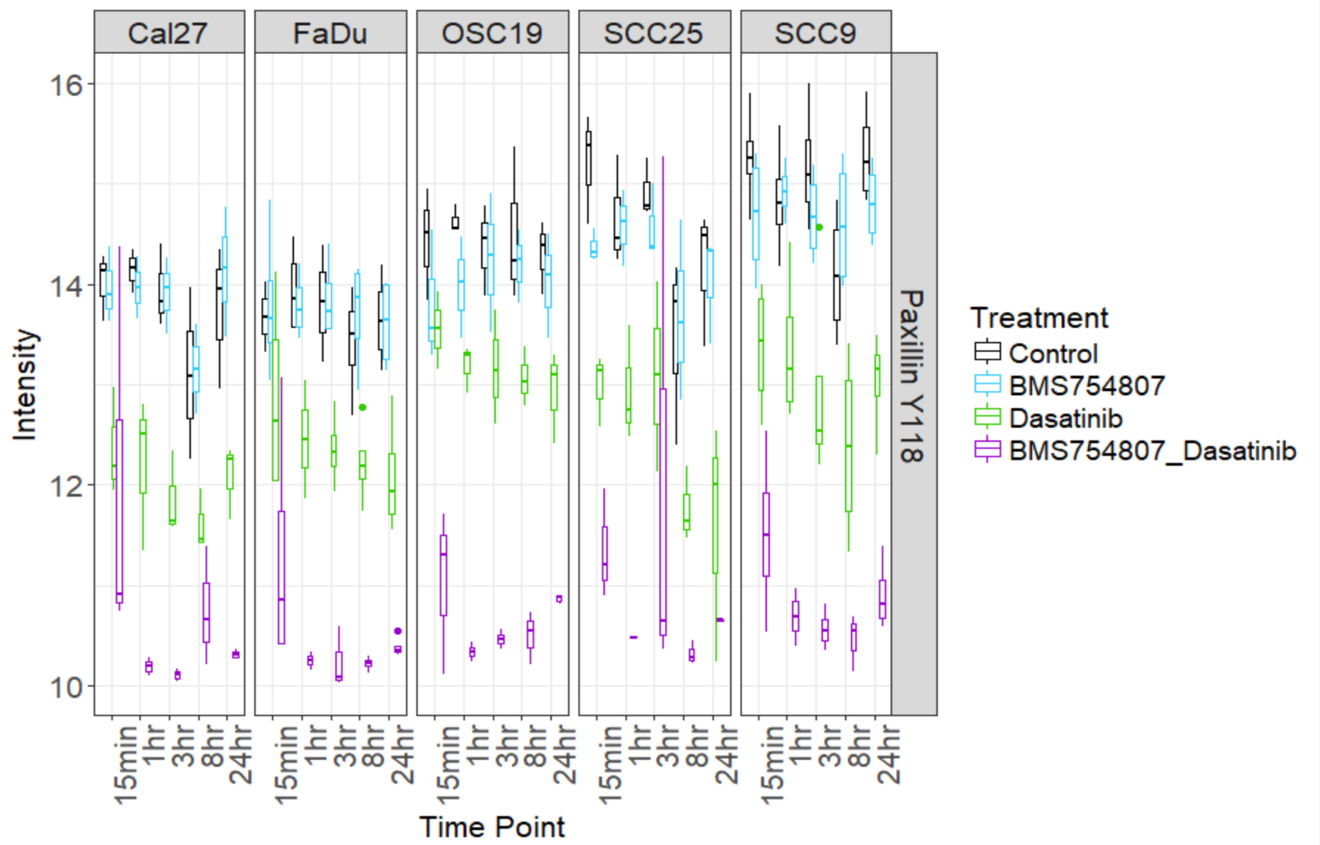

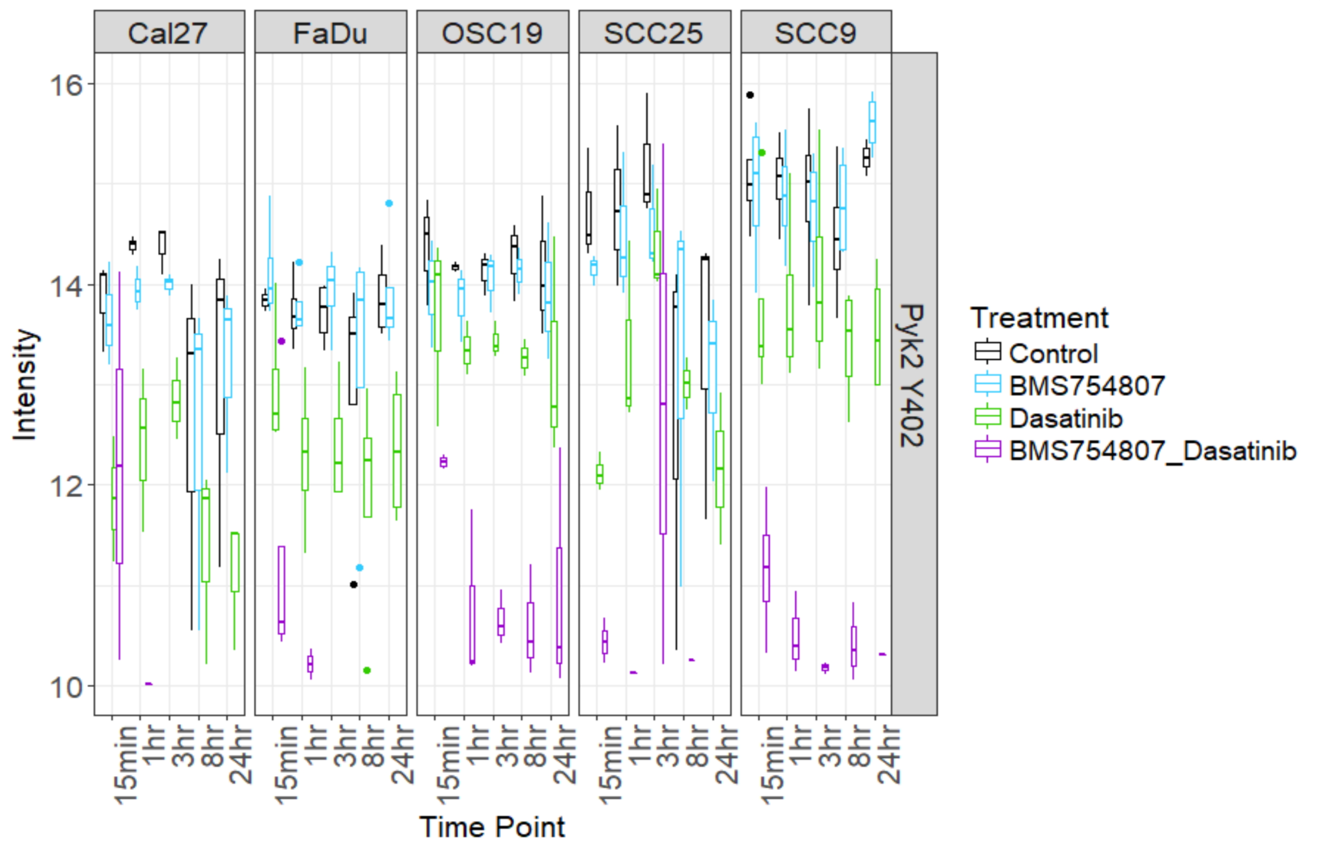

Supplemental Figure 2

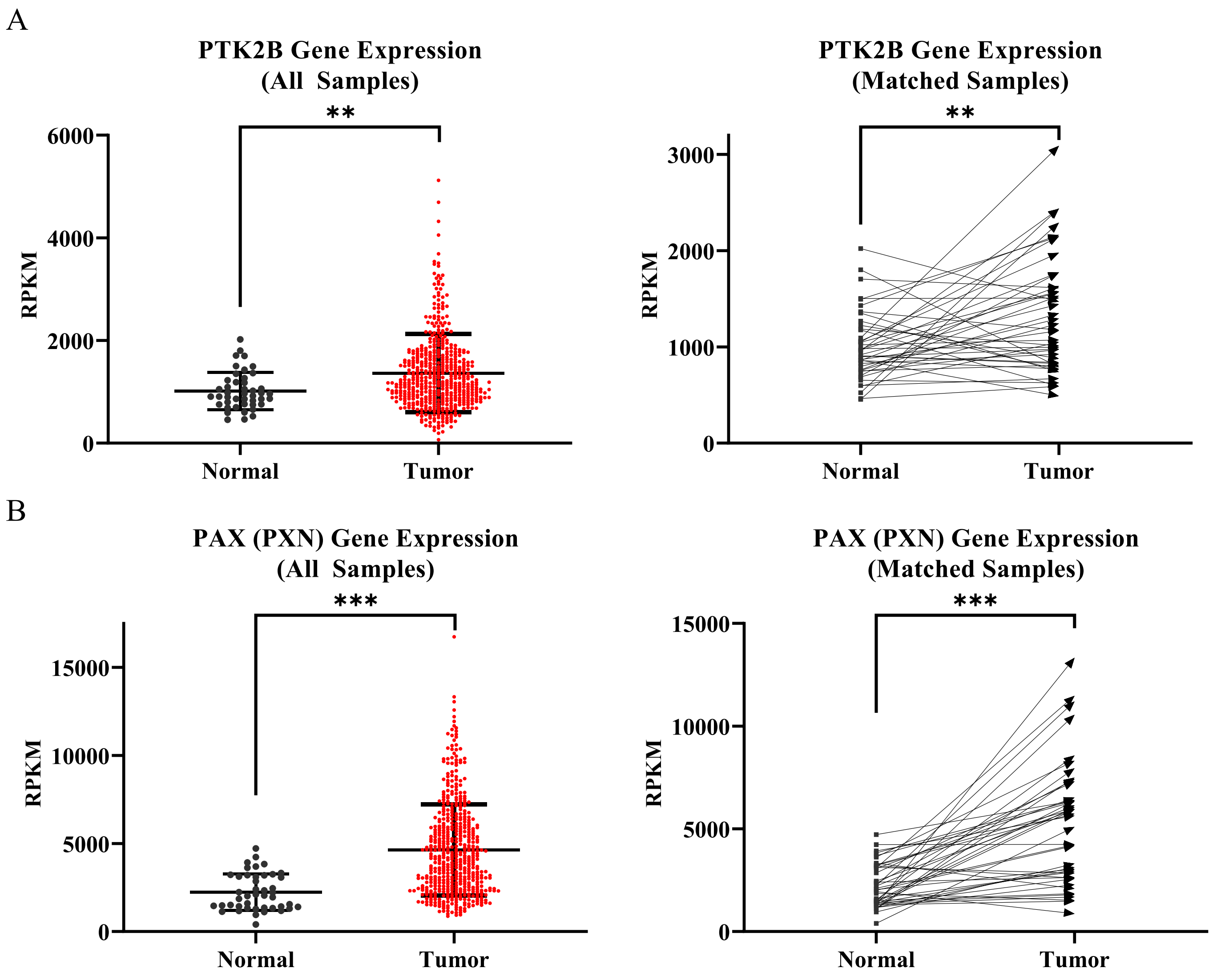

A. BMS-754807 - IGF1R

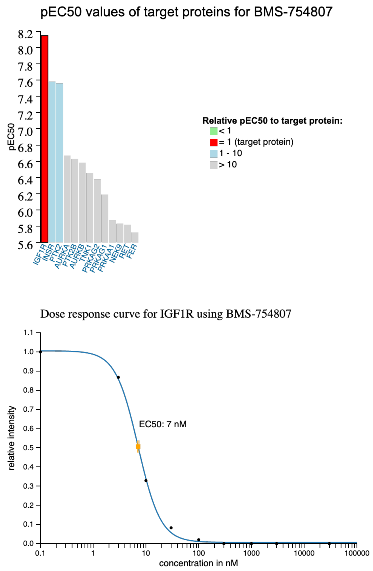

B. Dasatinib - Src

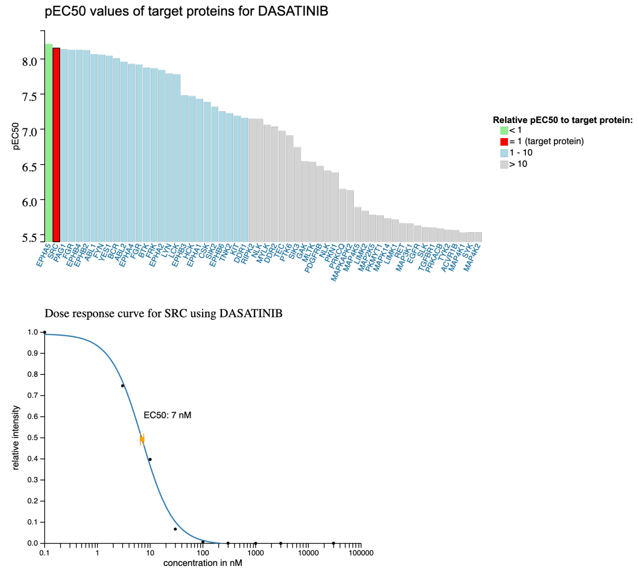

C. BMS-754807 - FAK

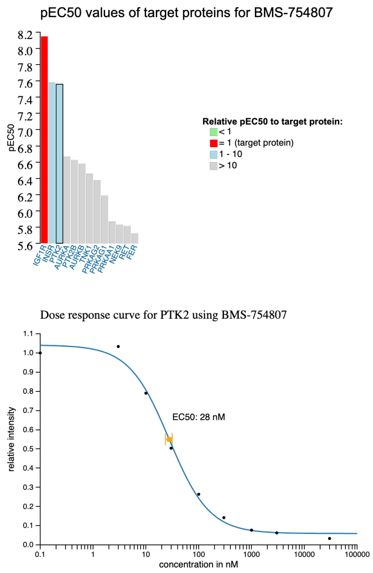

D. Linsitinib - IGF1R

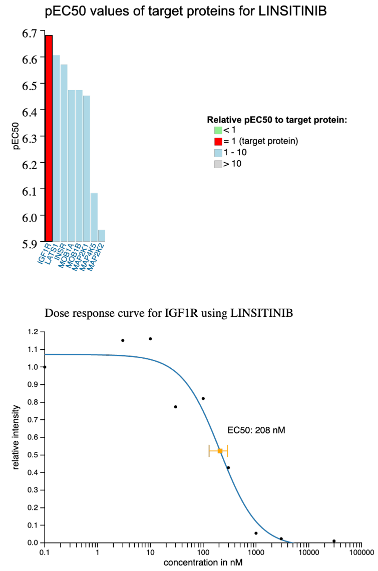

E. PF-562271 - FAK

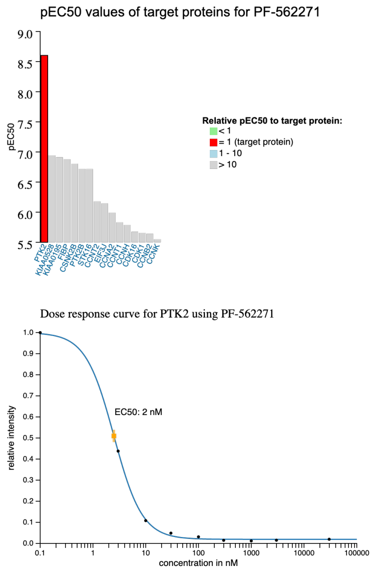

F. Defactinib - FAK

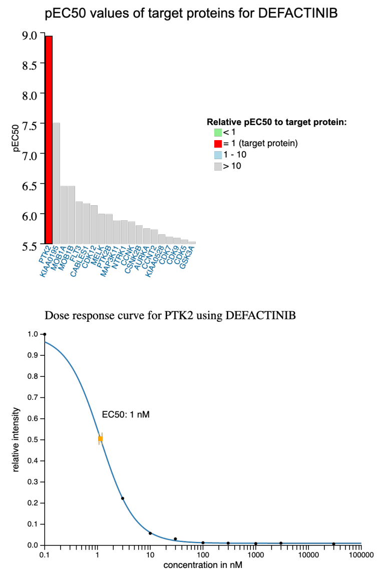

Supplemental Figure 3

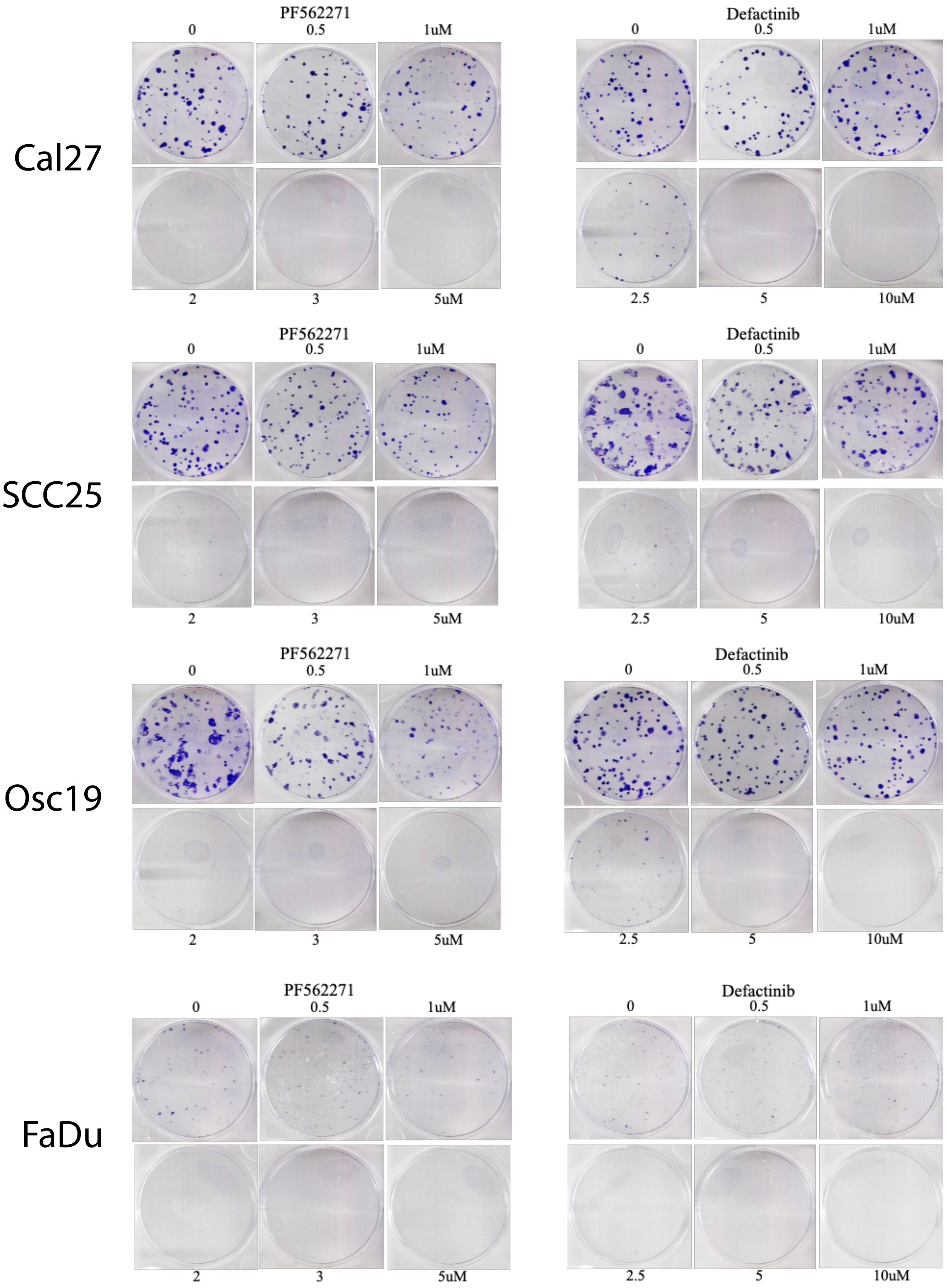

Supplemental Figure 4

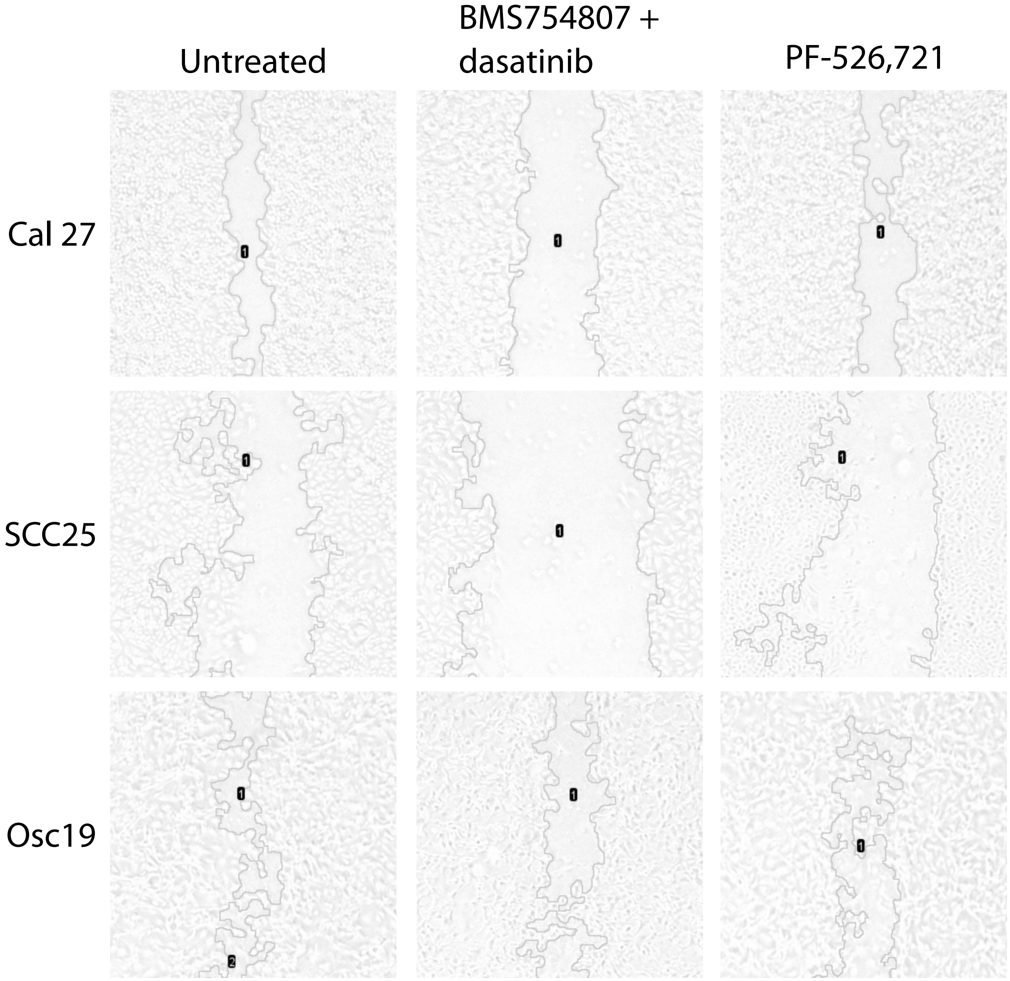

Supplemental Figure 5

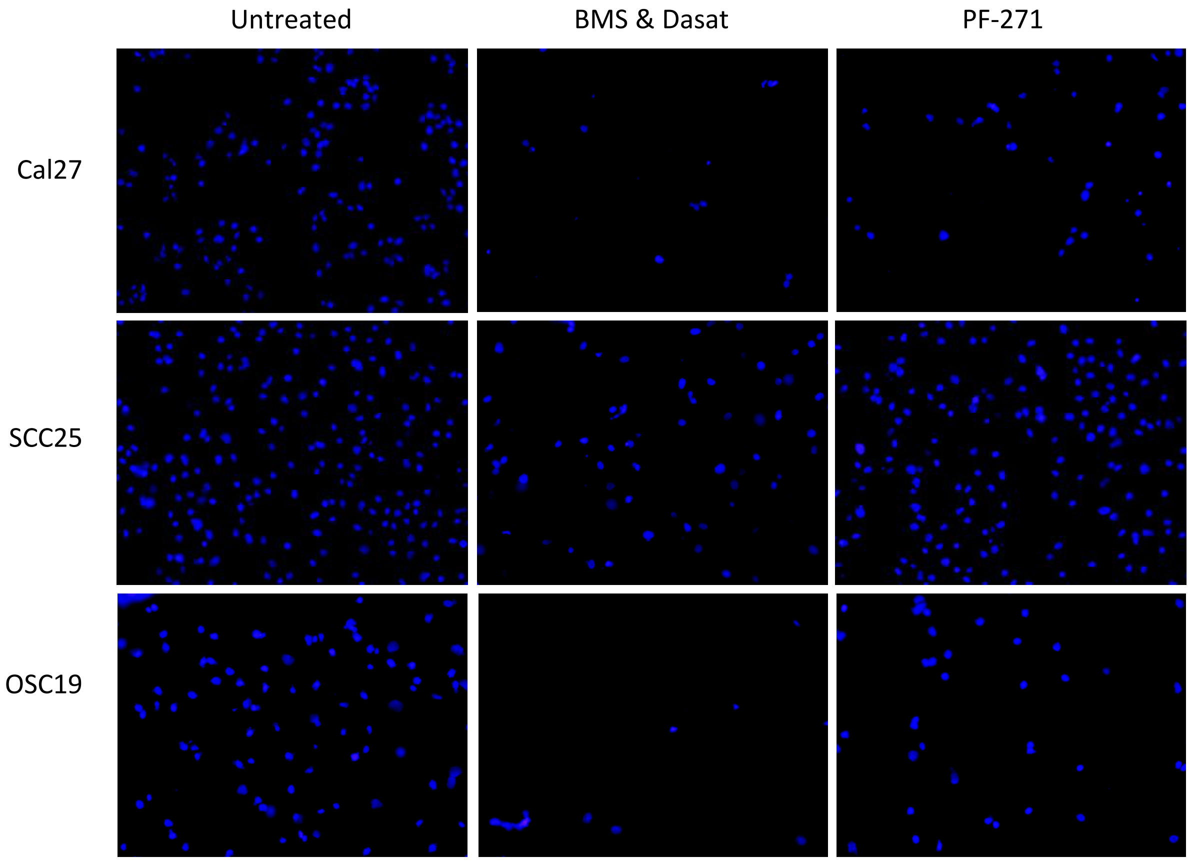

Supplemental Figure 6

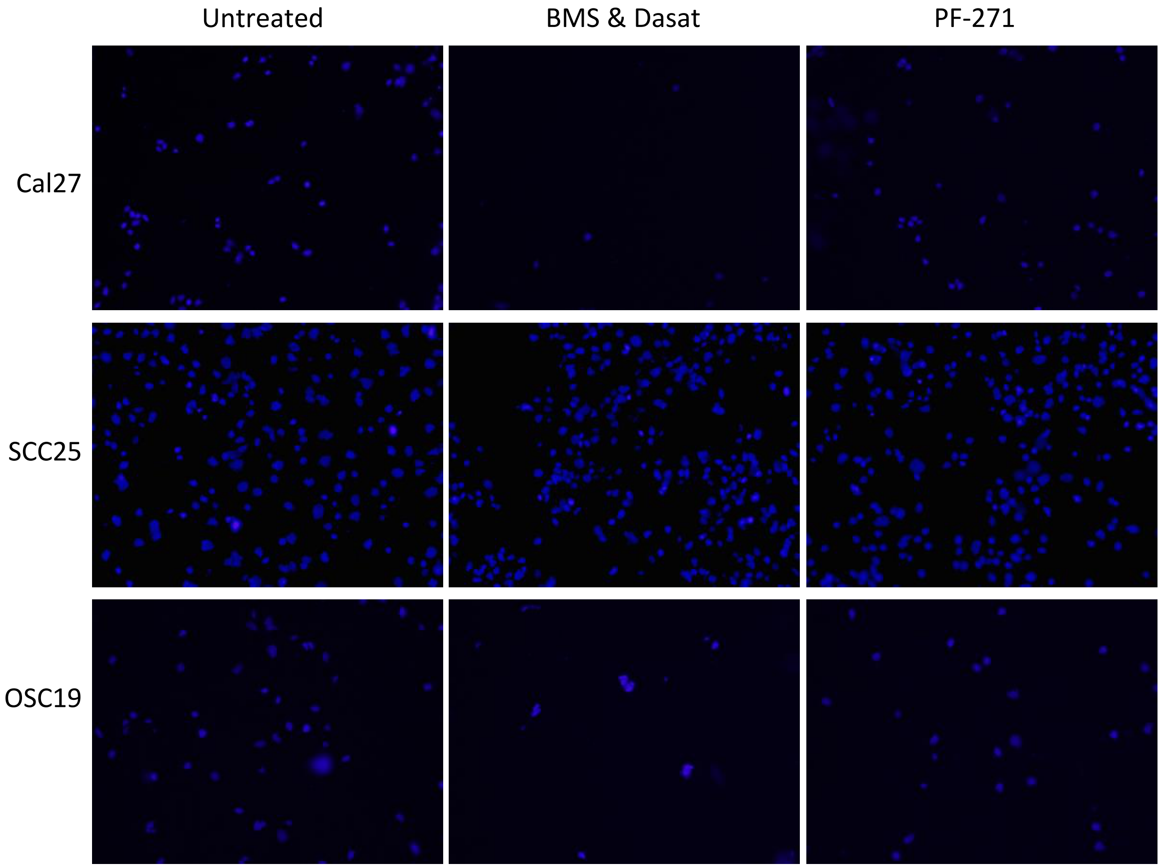

Supplemental Figure 7

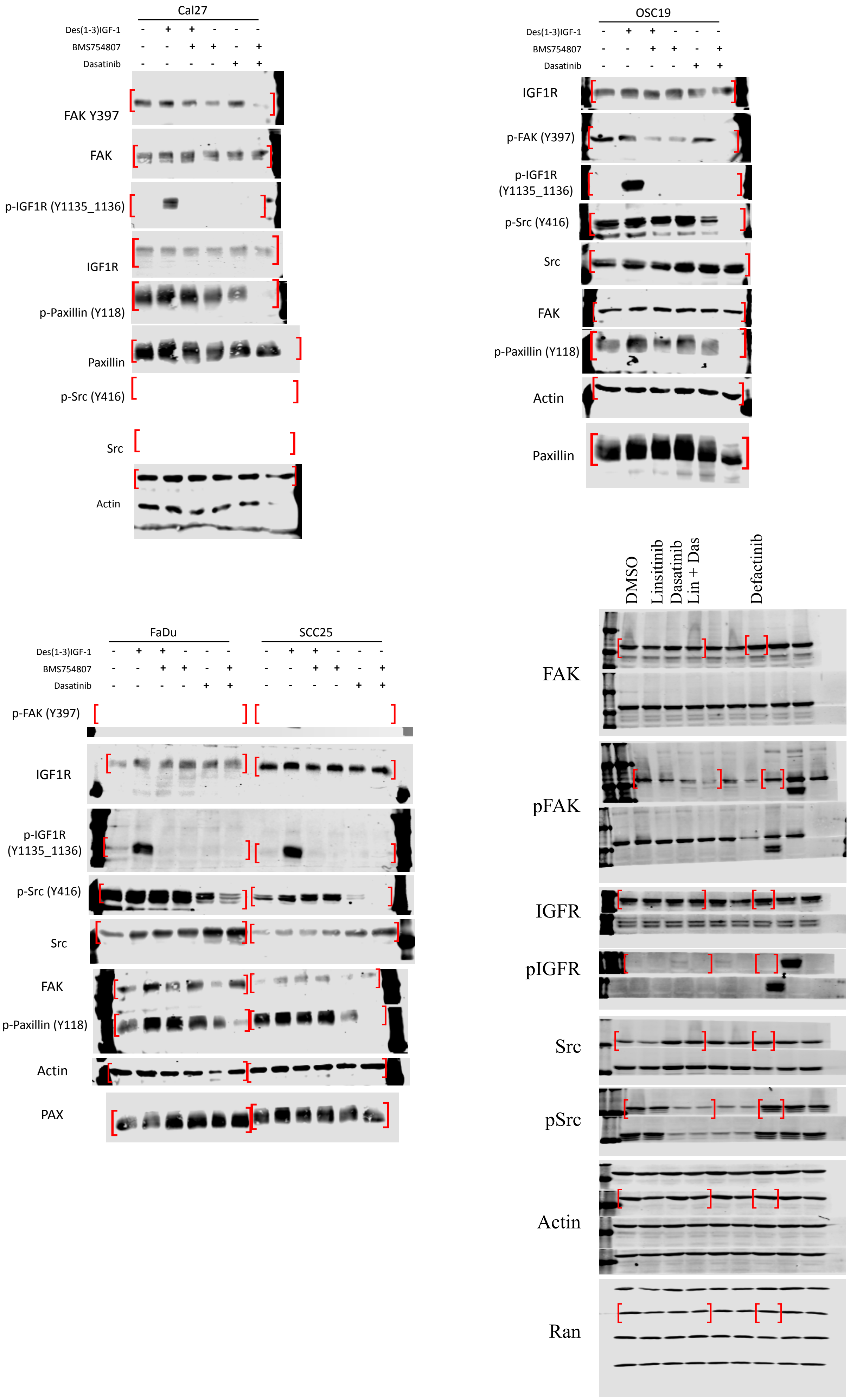

Supplemental Figure 8

Supplemental Table 1

Patient	Age	Sex	Anatomical Site	TNM stage
**1**	63	Male	post-cricoid/pyriform sinus	T4aN0M0
**2**	50	Male	posterior lateral oral tongue & tongue base	T3N2bM0
**3**	40	Male	transglottic (origin true vocal fold)	T4aN1M0
**4**	51	Male	floor of mouth & ventral oral tongue	T4aN2bM0
**5**	73	Male	lateral/ventral oral tongue	T2N0M0
**6**	84	Female	lateral oral tongue	T1N3bM0
